# Overexpression of Anti-müllerian Hormone Gene *in vivo* Affects Gonad Sex Differentiation in Undifferentiated Orange-Spotted Groupers (*Epinephelus coioides*)

**DOI:** 10.3389/fendo.2019.00210

**Published:** 2019-04-05

**Authors:** Yulong Han, Mi Zhao, Le Wang, Zeshu Yu, Jing Wang, Qi Yu, Ling Xiao, Mingwei Lu, Shuisheng Li, Yong Zhang, Haoran Lin

**Affiliations:** ^1^State Key Laboratory of Biocontrol, Guangdong Provincial Key Laboratory for Aquatic Economic Animals and Guangdong Provincial Engineering Technology Research Center for Healthy Breeding of Important Economic Fish, School of Life Sciences, Sun Yat-Sen University, Guangzhou, China; ^2^Department of Aquaculture, National Taiwan Ocean University, Keelung, Taiwan; ^3^Laboratory for Marine Fisheries Science and Food Production Processes, Qingdao National Laboratory for Marine Science and Technology, Qingdao, China; ^4^College of Ocean, Key Laboratory of Tropical Biological Resources of Ministry of Education, Hainan University, Haikou, China

**Keywords:** sex differentiation, *amh*, grouper, protogynous, hermaphroditic

## Abstract

Sex differentiation in teleost fishes occurs in response to sex determination signals, which induce the gonad to develop as either an ovary or testis. However, sex differentiation mechanisms in fishes are diverse, and information on gonad differentiation in sex changing fishes remains limited. The orange-spotted grouper (*Epinephelus coioides*) is a protogynous hermaphroditic fish that provides an ideal model for investigating gonad differentiation in vertebrates. In this study, Transcriptome data showed that expression levels of *amh* and *amhrII* in gonads were increased during sex differentiation. Then we investigated the effect of overexpression anti-Müllerian hormone (Amh) on gonad development in juvenile orange-spotted groupers. Expression levels of female-related genes and serum 17β-estradiol levels were decreased, while expression of male-related genes and serum 11-ketotestosterone levels were increased in fish fed with *amh*-plasmid. Overexpression of Amh was also promoted the spermatogonia proliferation and induced the development of male gonads in undifferentiated orange-spotted groupers, but that this male tendency was preceded by female differentiation. In summary, these results illustrated that Amh overexpression by *amh*-plasmid feeding induced male gonad development in undifferentiated groupers.

## Introduction

Sexual reproduction depends on the production of two types of gametes, which in turn rely on the specialized functions of the female and male gonads. Sex determination leads to a binary choice of gonad fate, to form either an ovary or a testis. The undifferentiated gonadal primordium is considered to be bipotential, and its subsequent development as either an ovary or testis in response to sex-determination signals is referred to as sex differentiation (1, 2).

The gonads of hermaphroditic, sex-changing fishes might be expected to have higher sexual plasticity than gonads of gonochoristic fishes; however, the unique and complex diversity of fish sex differentiation means that information on gonad differentiation in sex-changing fishes remains limited (1). Administration of low doses of exogenous sex steroid hormones (androgens or estrogens) leads to sex reversal in medaka, and this method has demonstrated that sex steroid hormones play an important role in gonad differentiation in a wide variety of fish species (3–5). Molecular biological studies have also shown that endogenous estrogen is a natural inducer of ovarian differentiation in gonochoristic teleosts (6–9). However, other hormones other than sex hormones can also influence sex differentiation in fish, including anti-Müllerian hormone (AMH), encoded by the *amh* gene (1). In female mammals, AMH acts by decreasing aromatase (cyp19a1)biosynthesis rather than by blocking enzyme activity (10). However, several of fishes show a negative correlation between *amh* and aromatase expression during the sex differentiation phase (11). Furthermore, higher levels of *amh* are correlated with lower *cyp19a1a* in zebrafish. These data indicate that *amh* is a candidate gene downregulating *cyp19a1a*, potentially leading to juvenile ovary-to-testis transformation (12). However, the role of *amh* during fish sex differentiation remains unclear.

Anti-Müllerian hormone (AMH) belongs to the transforming growth factor-beta superfamily, and has been implicated in male sex differentiation, female follicular development, and steroidogenesis in both sexes in mammals (13, 14). Although the “anti-Müllerian duct” effects of AMH have primarily been emphasized in higher vertebrates, *amh* has been identified in many teleost fish species that lack a Müllerian duct, such as Japanese eel (*Anguilla japonica*) (15), medaka (*Oryzias latipes*) (16), zebrafish (*Danio rerio*) (17), Japanese flounder (*Paralichthys olivaceus*) (18), and others (11), and has been shown to modulate the proliferation of spermatogonia (19). A duplicate copy of *amh* on the Y sex chromosome (*amhY*) is the master sex determinant in Atherinopsidae (*Odontesthes hatcheri*) (20) and Nile tilapia (*Oreochromis niloticus*) (21). The amh receptor type II gene (*amhrII*) has been identified as a sex-determining gene in Japanese pufferfish (*Takifugu rubripes*) (22), and *amhrII* mutations lead to sex reversal in medaka (*O. latipes*) (19, 23). These results suggest that *amh* may play a crucial role in sex differentiation in fish.

The orange-spotted grouper (*Epinephelus coioides*) is a protogynous hermaphroditic fish, which is economically valuable and widely cultured in southern Asia (24). These observations imply that ovarian differentiation may be the primary status in all sequential hermaphrodite species. In groupers, the gonads of all individuals initially differentiate directly into ovaries at approximately 47 days post-hatching (dph) (25), and most individuals initially mature as females at the age of 4–5 years, after which some individuals may undergo sex reversal from females to males (26–29), and primary oocytes were observed in the gonads of 1–3-year-old groupers (30). The orange-spotted grouper thus represents an ideal model for investigating gonad differentiation in vertebrates.

We previously showed that feeding groupers with an *amh*-overexpression plasmid induced female-to-male transition (31). However, the function of *amh* in the sex-differentiation process in groupers remains unknown. We therefore investigated the role of *amh* in sex differentiation in groupers using transcriptome sequencing technology to determine the *amh* expression patterns in gonads during the process of sex differentiation. Groupers were fed an *amh*-overexpression plasmid and the resulting expression patterns were investigated by immunohistochemistry (IHC), Western blotting, and real-time polymerase chain reaction (PCR) technology, to explore the potential role of *amh* in sex differentiation. The results will shed light on the molecular mechanisms regulating sex differentiation in marine fish and further our understanding of sex differentiation, and may ultimately provide guidance for innovations in grouper culture technology.

## Materials and Methods

### Animals and Tissue Sampling

Undifferentiated female groupers [75 days post-hatching (dph)] provide a useful animal model for investigating the regulation of sexual fate. Normally fed groupers were obtained from the Daya Bay Fisheries Development Center (Huizhou City, Guangdong Province, China), including fish from undifferentiated to complete differentiation stages. Gonad samples from normally fed groupers at 50, 80, 100, 130, 160, 180, and 240 days were also examined histologically.

Plasmid-feeding experiments were conducted over 125 consecutive days in orange-spotted groupers with undifferentiated gonads (age 80 days, body weight 22–35 g, body length 4.5–6.5 cm; Daya Bay Fisheries Development Center). All animal experiments were conducted in accordance with the guidelines and approval of the Animal Research and Ethics Committee of Sun Yat-Sen University.

### Transcriptome Sequencing

Transcriptome sequencing has been widely used to study gene expression changes, variable splicing, post-translational modifications, and mutations, etc., especially in different samples at different time points, or to examine changes in gene expression under different processing conditions (32). Gonad samples were obtained from normally fed groupers at 50, 90, 110, 180, and 240 dph, with two biological replicates per sample. Ten mRNA libraries were constructed from purified RNA of these gonad. mRNA-Seq (mRNA sequencing) services were carried out using Illumina HiSeq 2000 platform (Illumina Inc., San Diego, CA, USA) provided by BGI-Shenzhen, China.

### Packaging of Recombinant *amh* Plasmid for Feeding Experiments

An *amh*-overexpression plasmid was constructed by cloning the *amh* sequence into pcDNA4.0 (+) vector (Invitrogen, Carlsbad, CA, USA). The plasmids were then encapsulated in liposomes, which were formed by thin film hydration using DOTAP (Sigma-Aldrich, USA) and cholesterol (Sigma-Aldrich), based on a modification of our previous study (33). Briefly, DOTAP and cholesterol were heated to 70°C followed by heating in a water bath at 65°C for 2 h and ultrasonication at 25°C for 10 min. The mixture was then extruded through a 100-nm membrane filter and used to encapsulate empty pcDNA4.0 (+) plasmid or pcDNA4.0 (+)-*amh* plasmid by ultrasonication at 25°C for 30 min. The final concentration of the construct was 20 μg/μL.

The fish were divided randomly into two groups: an empty plasmid [empty pcDNA4.0 (+)] feeding group (*n* = 35) and an *amh*-plasmid [pcDNA4.0 (+)-*amh*] feeding group (*n* = 35). Both groups were cultured in separate seawater tanks. The empty plasmid group was fed a diet containing empty plasmid (1 mL/50 g food weight) and the *amh*-plasmid group was fed a diet mixed with *amh* plasmid (1 mL/50 g food weight). Seven individuals from each group were randomly sacrificed at 25, 50, 75, 100, and 125 days after treatment (dat), respectively and gonad tissues and serum samples were collected.

### RNA Isolation and Gene Expression Analysis

Total RNA was extracted from the gonads of orange-spotted groupers using TRIzol reagent (Invitrogen, America) and the cDNA samples were sent to BGI (Shenzhen, China) for sequencing. cDNA was produced from 1 μg total RNA from the feeding experiment samples using a Transcriptor First Strand cDNA Synthesis Kit (Roche, Swiss) and used as the template for subsequent real-time PCR analyses. The specific primers used in this study are listed in Table 1. The transcription levels of the target genes *amh, amhrII* (typeII anti-Müllerian hormone receptor), *sox9* (sex-determining region y-box 9), *cyp11b* (11beta-hydroxylase), *dmrt1* (doublesex and mab-3-related transcription factor 1), *hsd11b2* (11β-hydroxysteroid dehydrogenase type2), *cyp19a1a* (aromatase P450), and *foxl2* (ForkheadboxL2) were measured using a SYBR Green PCR Master Mix Kit (ABI, Vernon, CA, USA) with an ABI Real-Time PCR Fast System (ABI). The quantitative real-time PCR conditions were as follows: denaturation at 95°C for 5 min, followed by 40 cycles of 95°C for 15 s, 58°C for 20 s, 72°C for 20 s, and then 84°C for 10 s (fluorescent data collection). After amplification, the fluorescent data were converted to threshold cycle values (Ct). The relative abundance of mRNA transcripts was then evaluated using the formula: R = 2^−ΔΔCt^ (34).

**Table 1:** Nucleotide sequences of the primers used in this Study.

**Primers**	**Primers sequence (from 5′ to 3′)**
**PRIMERS FOR REAL-TIME PCR**	
*amh*-F	TGTTGGGAGCGACGGTGAACT
*amh*-R	TGCAGCGACTGACTCGTGAAA
*amhRII*-F	CAAATGCTGCGTGGGCTAT
*amhRII*-R	GAGGCTCTTCTGACTCTGTGGT
*dmrt1*-F	GCTGGAGTAGACTGCTTGTTT
*dmrt1*-R	CGACTGTGCGTCAGTATGAGC
*cyp11b*-F	TGTTGCCGTCTGACATCG
*cyp11b*-R	TCGCCACTCCTCACCGTTC
*sox9*-F	GCAATGCAGGCTCAGAATAG
*sox9*-R	GGTATCAAGGCAGTACCCAG
*cyp19a1a*-F	GGAGACATTGTGAGAGTCTGGATC
*cyp19a1a*-R	TGACAGGTACATCCAGGAAGAGTC
*foxl2*-F	CCACCGTACTCCTATGTCGC
*foxl2*-R	GTCTGATACTGTTCTGCCAAC
*β-actin*-F	ACCATCGGCAATGAGAGGTT
*β-actin*-R	ACATCTGCTGGAAGGTGGAC

### Gonad Histology

Gonads were dissected, fixed in Bouin's solution for 24 h at room temperature, dehydrated, and embedded in paraffin. The tissue blocks were sectioned at 5 μm using a microtome (RM2235; Leica, Germany) and stained with hematoxylin and eosin for gonad histology analysis.

### Western Blot and IHC Analysis

Rabbit anti-Amh and anti-Dmrt1 antibodies were produced by our laboratory previously (30, 31). Gonad tissues from plasmid-fed fish were examined by Western blotting using rabbit anti-Amh antibody (1:2,000 dilution; present study) and β-actin antibody (1:5,000 dilution; RRID: AB_2289225; Proteintech, USA) to detect the expression of *amh* and accumulation of the plasmid in the gonads.

IHC analyses were performed as described previously (35). Rabbit anti-Dmrt1 antibody (1:200 dilution) (30) was used to detect the emergence of spermatogonia in gonads from plasmid-fed fish. The samples were photographed under a light microscope (Nikon, Japan). The specific antibodies used in this study are listed in Table 2.

**Table 2:** Information of all antibodies list.

**Target antigen**	**Name of antibody**	**Manufacturer, catalog #,**	**Species raised in; monoclonal or polyclonal**	**Dilution used**	**RRID (required in revised MSs)**
Rat,mouse	ACTB antibody	Proteintech, #60008-1-lg	Mouse, monoclonal	3000	AB 2289225
IgG rabbit	Goat anti-rabbit IgG(H + L), HRP	Proteintech, #SA00001-2	Goat, polyclonal	3000	AB 2722564
IgG mouse	Goat anti-mouse IgG (H + L), HRP conjugate	Proteintech, #SA00001-1	Goat, polyclonal	3000	AB 2722565
Amh	Amh antibody	Our own preparation antibody	Rabbit, polyclonal	2000	
Dmrt1	Dmrt1 antibody	Our own preparation antibody	Rabbit, polyclonal	200	

### Serum 17β-estradiol (E_2_) and 11-ketotestosterone (11-KT) Assays

Blood samples were collected from the caudal vein of fish from each group. Serum samples were produced by centrifugation at 4°C and were stored at −20°C. Serum E_2_ and 11-KT levels were measured using enzyme immunoassay kits (Cayman Chemical Company, Ann Arbor, MI, USA).

### Terminal Deoxynucleotidyl Transferase dUTP Nick End Labeling (TUNEL) Assay During *amh* Plasmid Feeding

Apoptotic cells were detected using a TUNEL Apoptosis Detection Kit (Phygene, Fuzhou, China) according to the manufacturer's instructions. The samples were photographed under a light microscope (Nikon).

### Statistical Analyses

All data are expressed as the mean ± standard error of the mean (SEM). Statistical differences were estimated using Student's *t*-tests or one-way analysis of variance followed by Tukey's tests (SPSS Software, USA). A value of ^*^P < 0.05 or different letters indicate significant differences.

## Results

### Histology of Gonads in Normal Orange-Spotted Groupers From Undifferentiated to Fully Differentiated Stage

**Figure 1:**
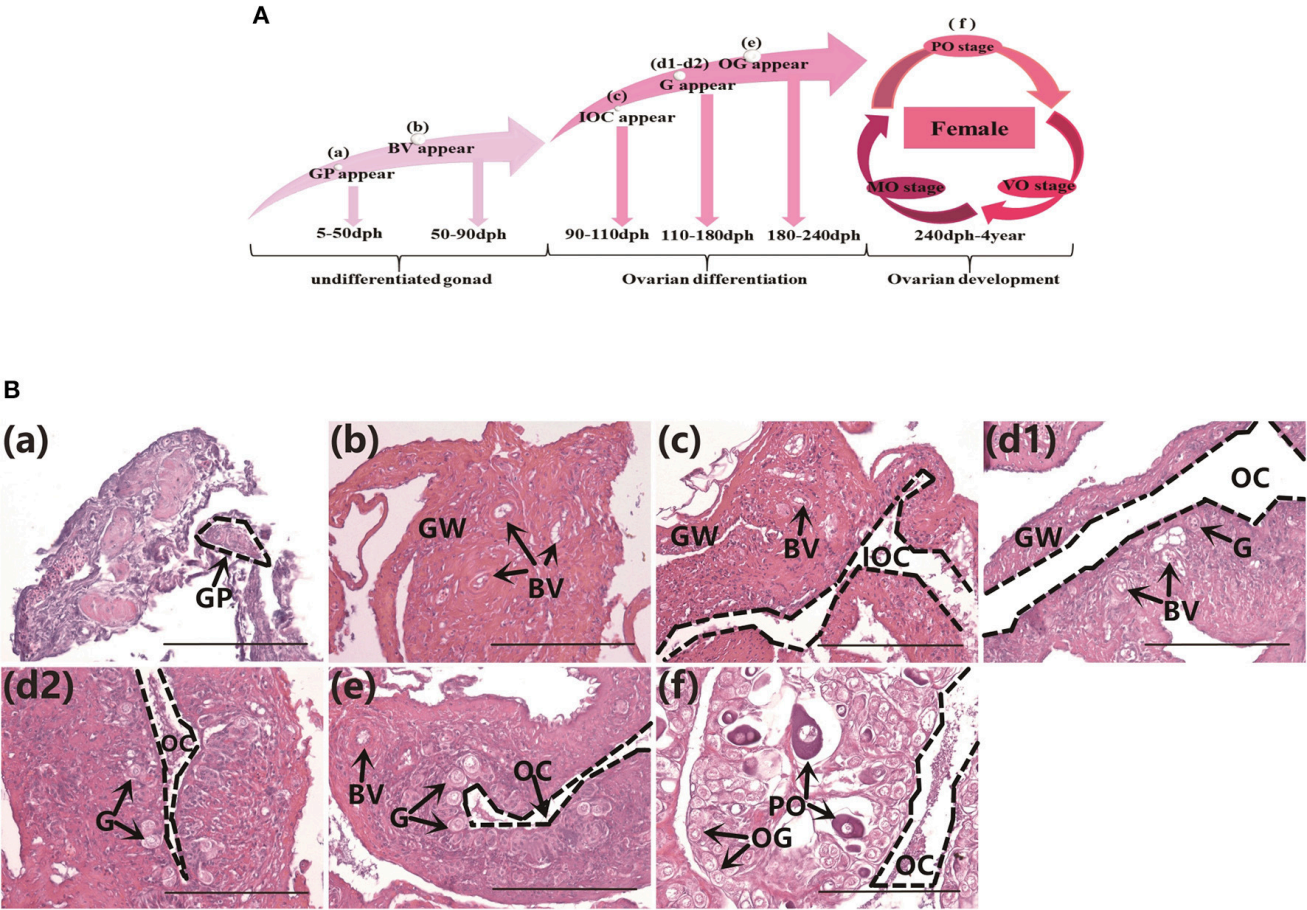
Gonad histology in normal orange-spotted groupers. **(A)** Gonad differentiation profiles in normal groupers. **(B)** Gonad histology in normal groupers at different developmental stages. **(Ba)** Gonad primordium appeared 50 days post-hatching (dph). **(Bb)** Blood vessels appeared 80 dph. **(Bc)** Initial ovarian cavity appeared 100 dph. **(Bd1,Bd2)** Gonia appeared 130–160 dph. **(Be)** Oogonia appeared 180 dph. **(Bf)** Primary oocytes appeared 240 dph. GP, gonad primordium; GW, gonad wall; BV, blood vessel; IOC, initial ovarian cavity; G, gonia; OC, ovarian cavity; OG, oogonium; PO, primary oocyte. Scale bars, 100 μm.

The gonad differentiation profile in groupers is shown in Figure 1A. We observed the gonads in normally-fed groupers from the undifferentiated stage to the completion of differentiation, at 50, 80, 100, 130, 160, 180, and 240 dph by histology. The gonad primordium had appeared at 50 dph (Figure 1Ba), blood vessels were present at 80 dph (Figure 1Bb), and the gonads had begun to differentiate by 100 dph and the initial ovarian cavity appeared (Figure 1Bc). The gonads continued to differentiate from 130–160 dph, the gonia appeared and continued to increase in number, and the ovarian cavity appeared (Figures 1Bd1,d2), the gonads continued to differentiate and oogonia appeared at 180 dph (Figure 1Be), and gonad differentiation was complete and primary oocytes were present by 240 dph (start of ovarian development stage) (Figure 1Bf). These results were consistent with those of previous studies (24).

### Identification of Genes Related to sex Differentiation by Transcriptome Sequencing

**Figure 2:**
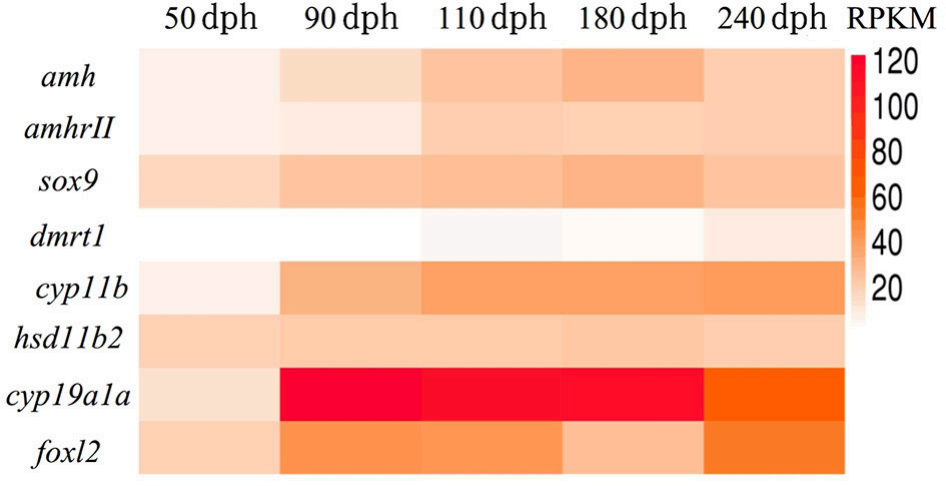
Transcriptome sequencing analysis of genes related to sex differentiation in normal orange-spotted grouper. All data were sampled from normal feeding grouper. RPKM (Reads Per Kilobase Million) of genes related to sex differentiation after transcriptome sequencing.

We previously revealed the approximate sex differentiation period in groupers (Yong Zhang & Qing Wang, unpublished observations). In the current study, we performed transcriptome analysis of gonads at different time intervals to screen for sex differentiation-related gene expression, an carried out transcriptome sequencing at 50, 90, 110, 180, and 240 days post-hatching (dph) from undifferentiated gonads to complete gonad differentiation. Regarding the expression levels of genes related to sex differentiation *in vivo*, expression levels of the female-related genes *cyp19a1a* and *foxl2* continued to rise, while the male-related genes *hsd11b2* and *dmrt1* remained at persistently low levels or were not expressed. Previous studies suggested that *amh* plays an important role in testis development (16, 21). However, we found that expression levels of both *amh* and *amhrII* were increased during sex differentiation in gonads (Figure 2). This suggested that *amh* expression may be essential for sex differentiation in gonads, but more in-depth studies were needed to determine the function of *amh* in the process of sex differentiation.

### Detection of Amh Levels by Western Blotting Following Plasmid Feeding

**Figure 3:**
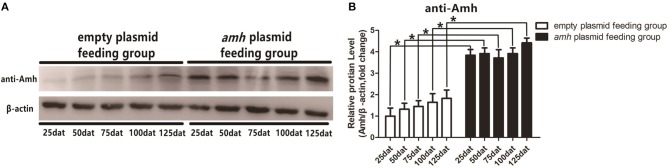
Western blot analysis of Amh expression in orange-spotted groupers following *amh*-plasmid feeding. **(A)** Amh expression was detected by western blot using rabbit anti-Amh antibody in gonads from groupers fed with *amh* plasmid. **(B)** Densitometric analysis of amh expression levels in gonads. Statistical differences were estimated using the Student's *t*-test analysis of variance followed by Tukey's tests (SPSS Software). *P < 0.05 indicate significant differences.

We detected Amh levels *in vivo* by Western blotting in groupers fed with *amh* plasmid, using β-actin as an internal control. Amh expression was significantly increased in the *amh*-plasmid group on all sampled days, but not in the empty-plasmid group. These results confirmed that feeding groupers with *amh* plasmid increased Amh protein expression in the gonad *in vivo* (Figures 3A,B).

### Amh Over-expression Changed the Direction of Sex Differentiation

**Figure 4:**
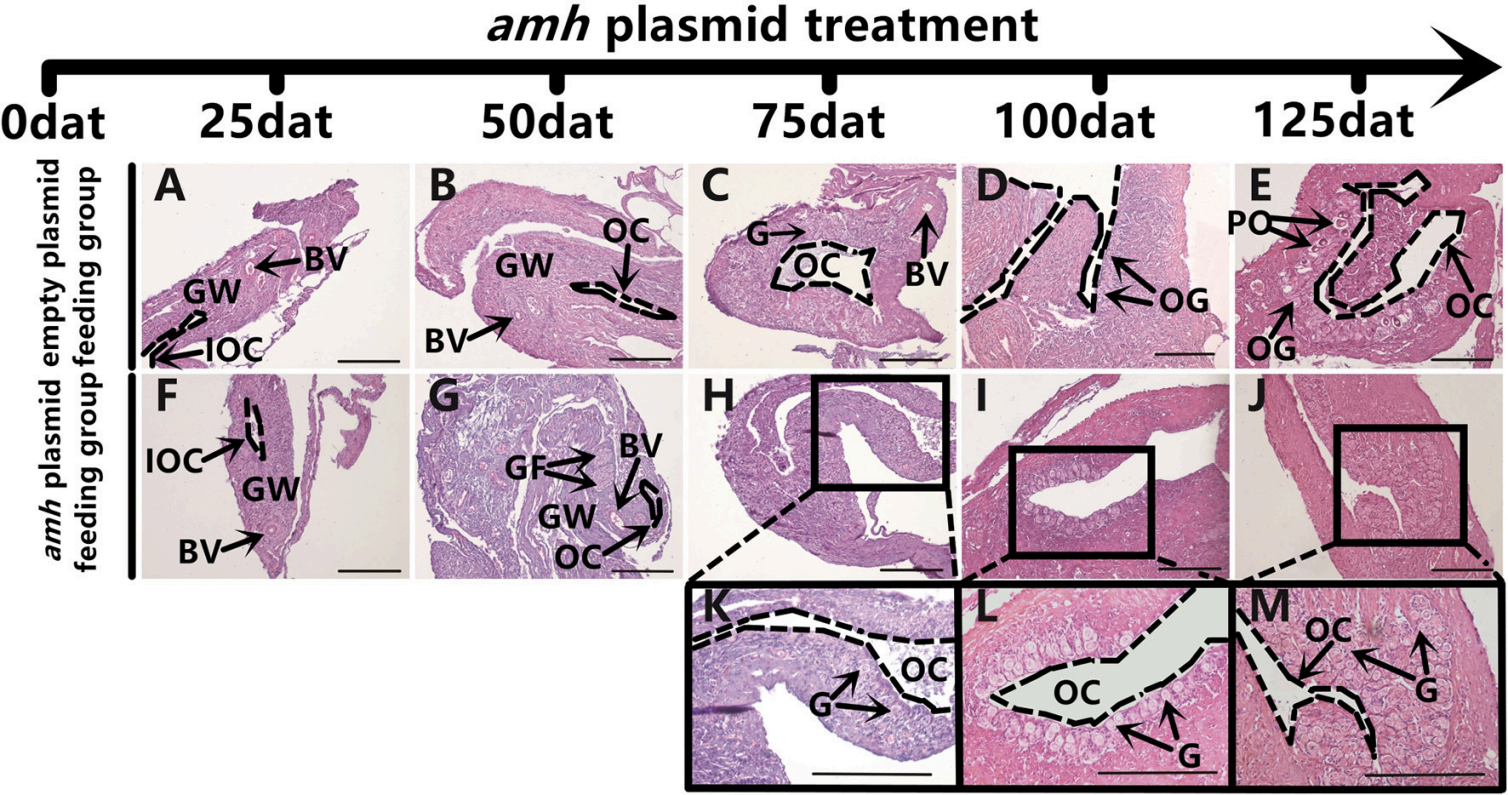
Gonad histology in orange-spotted groupers following *amh-*plasmid feeding. **(A–E)** Gonad histology in groupers fed empty plasmid at different sampling times. **(F–M)** Gonad histology in groupers fed *amh* plasmid at different sampling times. Panels **(K–M)** show high magnification views of boxed areas in **(H–J)**, respectively. GW, gonad wall; BV, blood vessel; IOC, initial ovarian cavity; GF, gonium formation; G, gonia; OC, ovarian cavity; OG, oogonium; PO, primary oocyte. Scale bars, 100 μm.

The gonadal status of each individual was examined by histology during the feeding experiments. The gonads developed normally in the empty-plasmid group from 25–50 dat (Figures 4A,B), and a few gonia appeared at 75 dat (Figure 4C), and some oogonia and primary oocytes appeared from 100–125 dat (Figures 4D,E). In contrast, gonad structure differed in the *amh*-plasmid group: the gonads developed normally at 25 dat (Figure 4F), but gonia formation was only observed in one fish at 50 dat, while no gonia were present in the other fish gonads (Figure 4G). About a third of gonads had a small amount gonia by 75 dat (Figures 4H,K), and about two-thirds had numerous gonia by 100 dat, while the remainder still had few gonia (Figures 4I,L). All gonads had abundant gonia by 125 dat (Figures 4J,M). In conclusion, normal gonads in empty-plasmid-fed fish developed abundant oogonia and primary oocytes, while *amh*-overexpressing fish (*amh*-plasmid-fed) developed a greater number of gonia, without primary oocytes (Table 3).

**Table 3:** Phenotype of gonadal changes in experimental feeding.

**Fish gonad**	**Empty plasmid feeding group**				***amh* plasmid feeding group**		
	**(*n* = 7)**				**(*n* = 7)**		
	**Female tendency**				**Female tendency**	**Male tendency**	
	**Normal ovary**	**Gonia appear**	**Oogonia appear**	**Primary oocyte appear**	**Normal ovary**	**A small amount of gonia appear**	**A great number of gonia appear**
25dat	7	0	0	0	7	0	0
50dat	7	0	0	0	6	1	0
75dat	5	2	0	0	5	2	0
100dat	0	2	5	0	2	5	0
125dat	0	0	5	2	0	0	7

### IHC Identification of Gonia in *amh*-Over-Expressing Fish

**Figure 5:**
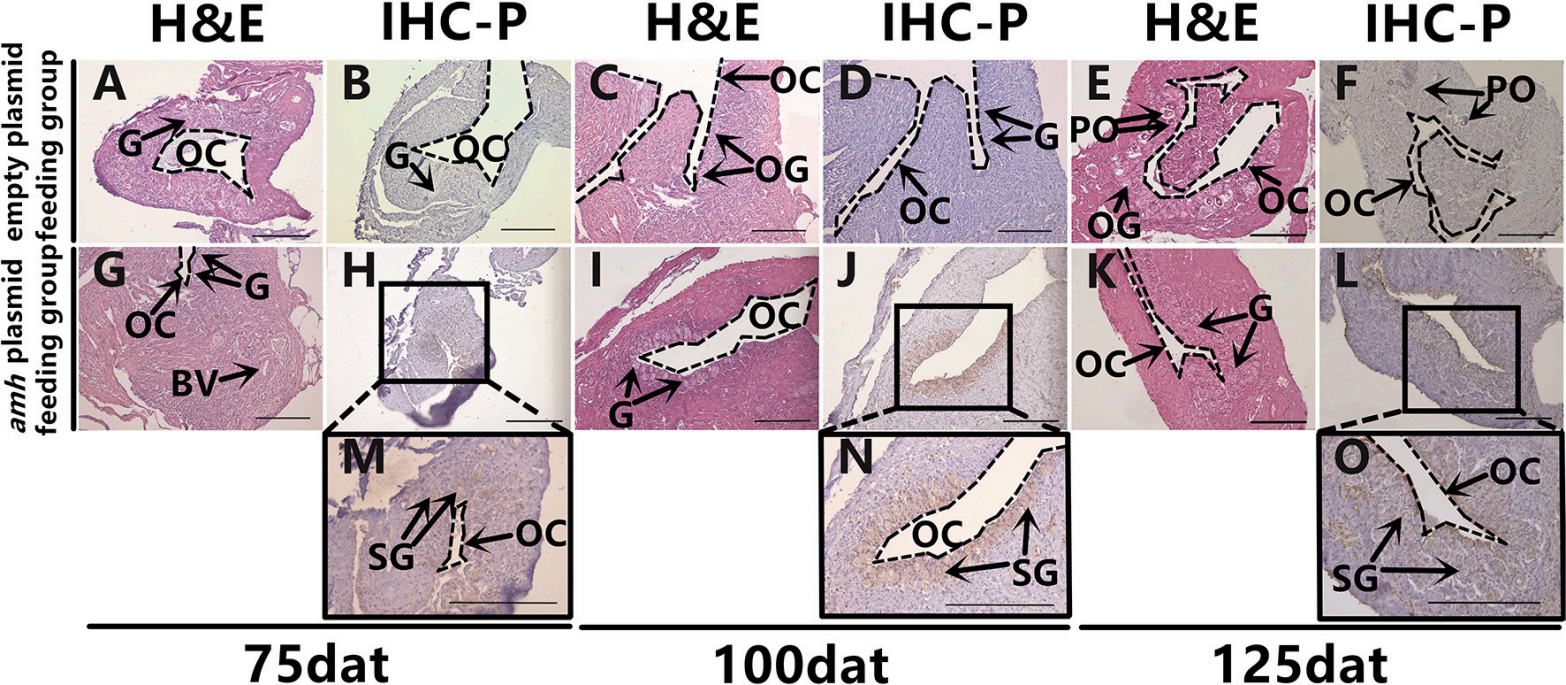
Dmrt1 signals detection in sex-differentiation process in orange-spotted grouper following *amh*-plasmid feeding. Hematoxylin and eosin staining of gonads sampled at different times **(A,C,E,G,I,K)**. IHC staining of gonads at different sampling times using rabbit anti-Dmrt1 antibody **(B,D,F,H,J,L–O)**. Panels **(M–O)** show high magnification views of boxed areas in **(H,J,L)**, respectively. BV, blood vessel; OC, ovarian cavity; G, gonia; OG, oogonium; PO, primary oocyte; SG, spermatogonia. Scale bars, 100 μm.

It is difficult to distinguish between oogonia and spermatogonia based on shape and size using light microscopy, and we therefore identified them by IHC. *Dmrt1* is a male-specific gene in groupers (36), and Dmrt1 protein only exists in spermatogonia and in primary and secondary spermatocytes in orange-spotted groupers (37). We therefore confirmed the presence of spermatogonia by IHC detection of Dmrt1 expression. Hematoxylin and eosin staining of gonads sampled at different times as a control (Figures 5A,C,E,G,I,K). No Dmrt1 signals were detected in the gonads in the empty-plasmid-fed group (Figures 5B,D,F), but a positive Dmrt1 signal was found in the *amh*-plasmid-fed group (Figures 5H,J,L). Furthermore, strong Dmrt1-positive signals were observed in the gonads from 75–125 dat (Figures 5H,J,L–O). These results demonstrated that *amh*-plasmid feeding increased the male tendency of gonad development, thus confirming the idea that Amh overexpression caused undifferentiated orange-spotted groupers to develop into males.

### Gene Expression and Serum Steroid Hormone Level Changes During *amh*-Plasmid Feeding

**Figure 6:**
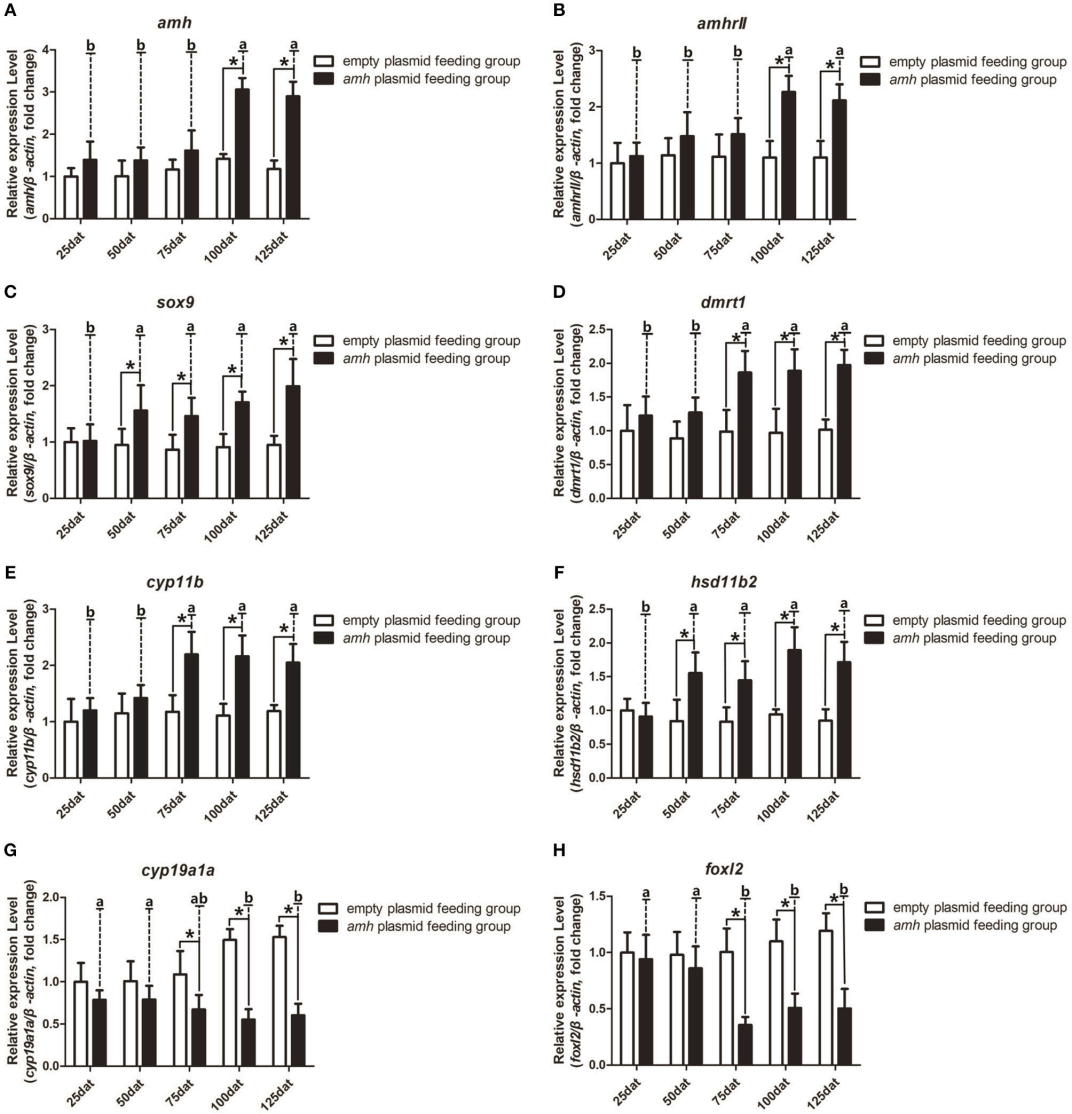
Expression profiles of key sex-differentiation genes in orange-spotted groupers following *amh-*plasmid feeding. **(A–F)** Gene expression of *amh* **(A)**, *amhrII* **(B)**, *sox9* **(C)**, *dmrt1* **(D)**, *cyp11b* **(E)**, *hsd11b2* **(F)**, *cyp19a1a* **(G)**, and *foxl2* **(H)** during *amh*-overexpression plasmid-induced sex reversal. β-actin was used as the internal control. Data expressed as the mean ± S.E.M from seven fish samples. Statistical differences were estimated using the Student's *t*-test or one-way analysis of variance followed by Tukey's tests (SPSS Software). *P < 0.05 or different letters indicate significant differences.

We also analyzed the expression profiles of key sex-differentiation genes during *amh*-plasmid feeding. *Amh* and *amhrII* expression levels were significantly increased from 100–125 dat (Figures 6A,B), *dmrt1* and *cyp11b* were significantly increased from 75–125 dat (Figures 6D,E), and *sox9* and *hsd11b2* were significantly increased from 50–125 dat (Figures 6C,F), while *cyp19a1a* and *foxl2* expression levels were significantly decreased from 75–125 dat (Figures 6G,H). These results indicated that *amh*-plasmid feeding stimulated male-pathway gene expression (*sox9, dmrt1, cyp11b*, and *hsd11b2*) and suppressed female-pathway gene expression (*cyp19a1a, foxl2*).

**Figure 7:**
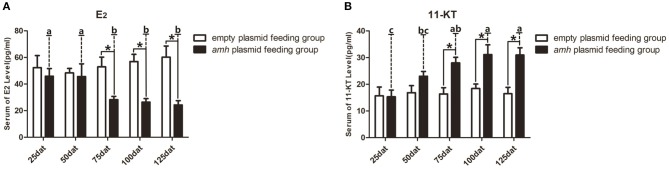
Serum sex steroid hormone levels in orange-spotted groupers following *amh*-plasmid feeding. **(A)** E_2_ levels in the empty-plasmid- and *amh*-plasmid-fed groups. **(B)** 11-KT levels in the empty-plasmid- and *amh*-plasmid-fed groups. Data expressed as the mean ± SEM from seven fish samples. Statistical differences were estimated using the Student's *t*-test or one-way analysis of variance followed by Tukey's tests (SPSS Software). *P < 0.05 or different letters indicate significant differences.

A balance between endogenous estrogens and androgens is known to be required to maintain the sexual identity of many fish species, and steroidogenesis plays a critical role in fish sex differentiation (30). *amh*-plasmid feeding significantly decreased serum E_2_ levels and significantly increased 11-KT levels from 75–125 dat compared with empty-plasmid-fed fish (Figures 7A,B). Estrogen is known to play an important role in maintaining ovarian development (30), and gradually reduced expression of female sex-differentiation genes in the gonads could lead to decreased E_2_ levels, resulting in degradation of female germ cells or disrupted ovarian development. In contrast, 11-KT plays a critical role in maintaining the testes, and high expression of male sex differentiation genes in the gonads could change the tendency of sex differentiation. The above results suggested that gonads may tend to develop in a male direction due to decreased estrogen levels and increased androgen levels in *amh*-plasmid-fed groupers.

### Apoptosis During Sex Differentiation in *amh*-plasmid-fed Groupers

**Figure 8:**
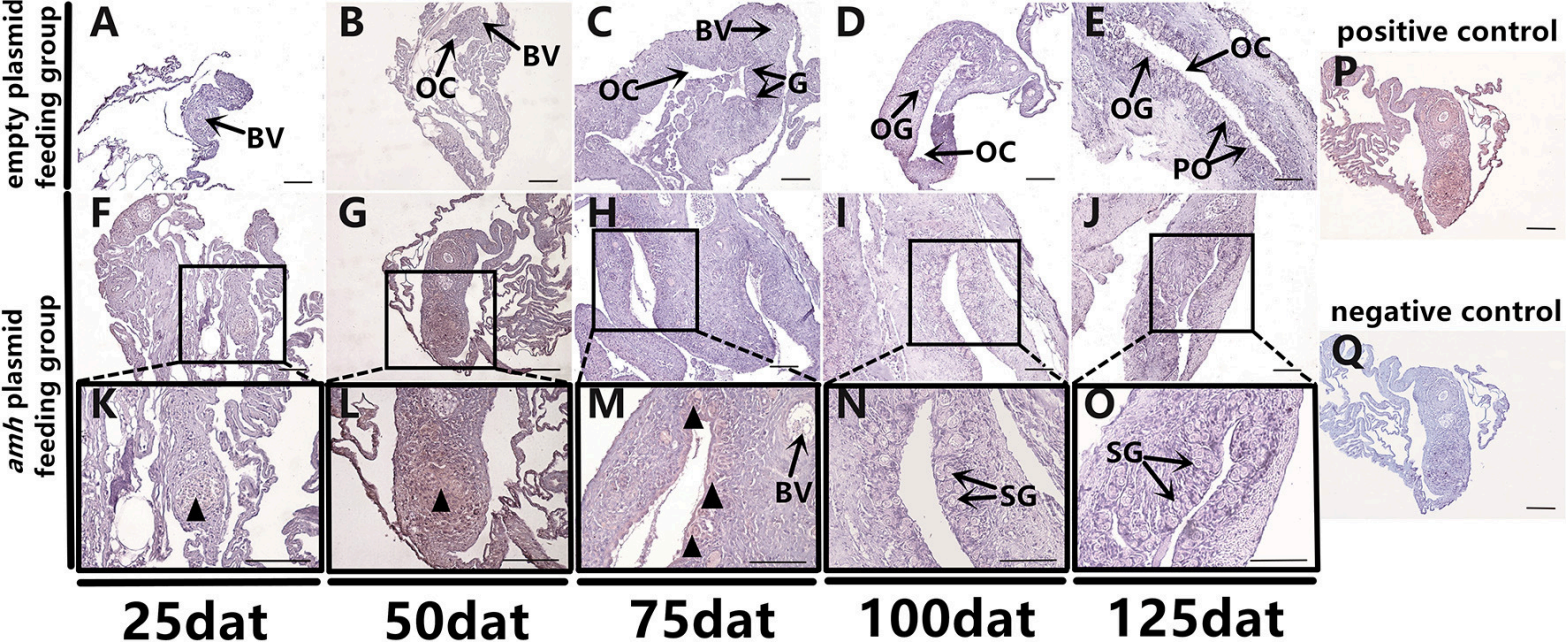
Apoptosis detection in sex-differentiation process in orange-spotted grouper following *amh*-plasmid feeding. Apoptosis in gonads sampled at different times. Black triangular arrow indicates apoptotic signal. **(A–E)** Gonad apoptosis in empty-plasmid-fed groupers at different sampling times. **(F–O)** Gonad apoptosis in *amh-*plasmid-fed groupers at different sampling times. **(P,Q)** Positive and negative controls. Panels **(K–O)** Show high magnification views of the boxed areas in **(F–J)** respectively. BV, blood vessel; OC, ovarian cavity; G, gonia; OG, oogonium; PO, primary oocyte; SG, spermatogonia. Scale bars, 50 μm.

Groupers are well-known to be protogynous fish, with primary oocytes observed in the gonads of 1–3-year-old orange-spotted groupers (30). Most orange-spotted groupers first mature as females at 4–5 years old, and some may then undergo sex reversal from females to males (26–29). We aimed to determine if *amh*-plasmid feeding before sex differentiation induced the gonads to develop directly into males, or if the gonads first developed as females before showing male differentiation. We therefore detected gonadal apoptosis in *amh*-plasmid-fed fish during differentiation. No apoptosis was detected in the empty plasmid group from 25–125 dat (Figures 8A–E), while gonads in the *amh*-plasmid-fed group showed an apoptotic signal at 25 dat (Figures 8F,K), which increased from 50–75 dat (Figures 8G,H,L,M), but was absent from 100–125 dat (Figures 8I,J,N,O). There are also positive and negative controls (Figures 8P,Q). These results suggested that Amh overexpression induced the development of male gonads in undifferentiated orange-spotted groupers, but that this male tendency was preceded by female differentiation (Table 4).

**Table 4:** Apoptosis of gonads in the normal-fed and *amh* pasimid-fed groupers.

**Fish gonad**	**Empty plasmid feeding group**		***amh* plasmid feeding group**	
	**(*n* = 7)**		**(*n* = 7)**	
	**Apoptotic signal**	**No apoptotic signal**	**Apoptotic signal**	**No apoptotic signal**
25dat	0	7	1	6
50dat	0	7	6	1
75dat	0	7	5	2
100dat	0	7	0	7
125dat	0	7	0	7

## Discussion

In this study, we observed the gonads in normally-fed groupers from the undifferentiated stage to the completion of differentiation (Figure 1). These results were consistent with those of previous studies (24). Despite the fact that hermaphroditism is widespread in many fish lineages (38), but only studies on sex differences have been conducted in only a few hermaphrodites. As the grouper is a protogynous hermaphroditic fish, therefore, the expression level of *amh* in the gonad differentiation of grouper is different from the gonochoristic fish.

In gonochoristic fish, elevated *amh* levels in male gonads are a common feature in fish during sex differentiation phase. That indicates an involvement of *amh* in male sex differentiation. Therefore, in recent gene expression studies elevated *amh* levels has been used as an indirect method for sexing developing gonads from Japanese flounder (39), Southern flounder (40), Atlantic cod (41), Nile tilapia (42), and pejerrey (43, 44). The divergent *amh* expression patterns in different species reflect the different modes of sex differentiation in fish (11). Through transcriptome sequencing data, we found that expression levels of both *amh* and *amhrII* were increased during sex differentiation in gonads (Figure 2). We speculate that although the expression of *amh* in the ovary is much lower than that in the testis, *amh* also plays a role in regulating ovarian development, but the role of *amh* in ovary require a more in-depth study. Besides, through transcriptome sequencing data, we also found regarding the expression levels of the female-related genes *cyp19a1a* and *foxl2* continued to rise, while the male-related genes *hsd11b2* and *dmrt1* remained at persistently low levels or were not expressed.

Despite the diversity of sex-differentiation processes among fishes, ovarian differentiation can be directed by estrogens and their synthetic aromatase enzymes in most teleosts. P450 aromatase (*cyp19a1a*) is the most important steroidogenic enzyme for ovarian differentiation and plays an essential role in the production of E_2_, which is believed to be the major sex hormone for inducing and maintaining ovarian development in fish (4). In female mammals, AMH acts by decreasing aromatase (cyp19a1)biosynthesis rather than by blocking enzyme activity (10). However, several of fishes show a negative correlation between *amh* and aromatase expression during the sex differentiation phase (11). An inverse association between *cyp19a1a* and *amh* expression has been reported in numerous fishes, including Japanese flounder (*P. olivaceus*) (45), rainbow trout (*Oncorhynchus mykiss*) (46), zebrafish (*D. rerio*) (12), southern flounder (*Paralichthys lethostigma*) (40), and pejerrey (*Odontesthes bonariensis*) (43). Although a causal relationship cannot be established from these studies, the results suggest that *amh* is a candidate gene down-regulating *cyp19a* (12). We also found an inverse association between *amh* and *cyp19a1a* expression in our current study, and *amh* overexpression *in vivo* inhibited *cyp19a1a* gene expression and led to significantly decreased serum E_2_; however, it remains unclear if *amh* directly downregulates *cyp19a1a* expression. We also analyzed the expression profiles of other key sex-differentiation-related genes during gonad differentiation in groupers. Feeding with an *amh* plasmid stimulated male-pathway gene expression (*sox9, dmrt1, cyp11b*, and *hsd11b2*) and suppressed female-pathway gene expression (*cyp19a1a, foxl2*), and induced a male tendency during sex differentiation in undifferentiated groupers. These results suggested that *amh* may have an important role in sex reversal in protogynous fish, by suppressing gonadal aromatase expression and/or activating a male-specific expression pathway.

The *AMH* gene is widely found in the gonads in invertebrates and vertebrates, and plays an important regulatory role in gonad development in vertebrates, and AMH is important for primordial follicle recruitment and folliculogenesis in mammals (47, 48). In contrast to mammals however, *amh* expression differs among different species of fish and its role in the ovary in teleosts, especially protogynous hermaphroditic species, remains largely unknown. A previous study showed that Amh influenced germ cell number, and high expression levels during early male development suggested that it played a role in male sex determination (11). Furthermore, feeding groupers with an *amh*-overexpression plasmid induced female-to-male transition (31). No sex chromosomes have yet been identified in groupers, which may be due to the special sex differentiation mechanism of female prematuration of hermaphrodites, and further sex differentiation research at the gene level is still required (49). Increasing evidence suggests that the *amh* gene plays an indispensable role in the process of fish sex differentiation (20, 50), though its involvement in early sex differentiation in the orange-spotted grouper remains unclear. The current results showed that fish fed an *amh* plasmid developed a male tendency, thus supporting the idea that Amh overexpression could induce male sex in undifferentiated orange-spotted groupers, while male tendency was preceded by female differentiation. However, further studies are needed to establish the time required to achieve complete male sex differentiation, and whether withdrawal of *amh* supplementation would lead to reversion to ovaries.

In summary, *amh* played an important role in sex differentiation in groupers, including orange-spotted groupers that Amh overexpression caused undifferentiated orange-spotted groupers to develop into males. A molecular-level understanding of sex differentiation, including the upstream factors responsible for initiating this process, would enable the development of efficient, low cost genetic tools for controlling sex ratios, thereby avoiding the use of steroid treatments, which pose significant environmental contamination risks. However, further studies are needed to clarify the mechanism and regulatory networks of *amh* in the process of sex differentiation in bony fish.

## Author Contributions

YH, MZ, and YZ contributed conception and design of the study. YH and LW organized the database. ZY, JW, and QY performed the statistical analysis. ML provides the packaging of recombinant *amh* plasmid. YH wrote the first draft of the manuscript. MZ, LX, SL, YZ, and HL wrote sections of the manuscript. All authors contributed to manuscript revision, read, and approved the submitted version.

### Conflict of Interest Statement

The authors declare that the research was conducted in the absence of any commercial or financial relationships that could be construed as a potential conflict of interest.
